# Dynamic Immune Landscape and VZV-Specific T Cell Responses in Patients With Herpes Zoster and Postherpetic Neuralgia

**DOI:** 10.3389/fimmu.2022.887892

**Published:** 2022-06-01

**Authors:** Qiao Peng, Xuejiao Guo, Yang Luo, Guocan Wang, Lingyu Zhong, Jiamin Zhu, Yunze Li, Xun Zeng, Zhiying Feng

**Affiliations:** ^1^ State Key Laboratory for Diagnosis and Treatment of Infectious Diseases, National Clinical Research Center for Infectious Diseases, National Medical Center for Infectious Diseases, Collaborative Innovation Center for Diagnosis and Treatment of Infectious Diseases, The First Affiliated Hospital, Zhejiang University School of Medicine, Hangzhou, China; ^2^ Department of Pain Medicine, The First Affiliated Hospital, Zhejiang University School of Medicine, Hangzhou, China; ^3^ Center for Stem Cell and Regenerative Medicine, Zhejiang University School of Medicine, Hangzhou, China

**Keywords:** varicella-zoster virus, herpes zoster, postherpetic neuralgia, CyTOF, VZV-specific T cells

## Abstract

**Objectives:**

Varicella-zoster virus (VZV) can induce herpes zoster (HZ) and postherpetic neuralgia (PHN). Immune cells play an important role in regulating HZ and PHN pathogenesis, but the dynamic immune profiles and molecular mechanisms remain unclear. This study aimed to screen dynamic immune signatures during HZ progression and elucidate the mechanism of VZV-specific T cells in PHN.

**Methods:**

We used cytometry by time-of-flight (CyTOF) to analyze peripheral blood mononuclear cells (PBMC) samples from 45 patients with HZ and eight age-sex-matched healthy controls, eight PHN samples and seven non-PHN samples. Correlations between the immune subsets and clinical pain-related scores were performed. Further, the characteristics of VZV-specific T cells between PHN and non-PHN patients were evaluated by VZV peptide pools stimulation. The expression level of cytokines, including granzyme B, interleukin (IL)-2, interferon (IFN)-γ, and tumor necrosis factor (TNF)-α was performed *via* cytometric bead array. Finally, we analyzed the alteration of Ca^2+^ signals in dorsal root ganglion (DRG)-derived cells after TNF-α stimulation.

**Results:**

We investigated the dynamic characteristics of the immune landscape of peripheral blood samples of patients with HZ and PHN, and depicted two major dynamic signatures in NK, CD4^+^ and CD8^+^ T subsets in patients with HZ, which closely correlated with clinical pain-related scores. The frequency of PD-1^+^CD4^+^ T cells, VZV-specific PD-1^+^CD4^+^ T cells, and the amount of TNF-α produced by VZV-specific T cells were higher in patients with PHN than without PHN. Furthermore, we showed that TNF-α could induce calcium influx in DRG-derived cells in a dose-dependent manner.

**Conclusions:**

Our results profiled the dynamic signatures of immune cells in patients with HZ and highlighted the important role of VZV-specific T cells in the pathogenesis of PHN.

## Introduction

Herpes zoster (HZ), often known as shingles, is caused by the reactivation of varicella-zoster virus (VZV), a human host-restricted α-herpes virus with a high incidence worldwide, particularly in immunocompromised or elderly individuals (1, 2). Postherpetic neuralgia (PHN) is defined as persistent pain for at least 3 months after HZ initiation and is the most common and debilitating complication following HZ in approximately 20% of patients (3, 4). PHN may cause physical disability, psychological depression, and financial burden that reduce the quality of life of patients (5). One of the most common syndromes for HZ and PHN is pain (6). However, current clinical pain indicators, such as rating scales and symptom-based questionnaires, have low sensitivity and reliability and fail to point out the underlying pathogenesis of pain (7, 8). Therefore, more accurate, sensitive, and objective pain indicators are required in the clinic. In addition, current therapies for both HZ and PHN, including anti-virus treatment, medication, nerve blocks, and radiofrequency, have many adverse effects or limited efficacy (9, 10). Thus, investigating the disease pathogenesis to explore the new targets for efficient therapies of HZ and PHN is urgent and important, which may improve the efficacy of HZ therapy and prevent the incidence of PHN from acute HZ.

The immune system is crucial in VZV infection. Natural killer (NK) cells and monocytes, vital components of the innate immune system, are significantly elevated in HZ patients (11). NK cell expansion is associated with more pronounced inflammation during HZ (11), and monocytes produce IL-6, IL-8, IL-12, IFN-γ, and TNF-α in response to VZV infection (12, 13). Apart from the innate immune system, adaptive immunity, especially T cell-mediated response, plays a definitive role in host defense against VZV infection. T cell xenografts in severe combined immunodeficiency (SCID) mice *in vivo* or tonsil T cells *in vitro* have validated that T cells can be infected with VZV. Furthermore, VZV-infected T cells are fully permissive to VZV replication and release (10, 14). The VZV-infected T cells predominantly display CD69^+^CD45RA^-^, an activation and memory phenotype (14). Part of them also express cutaneous leukocyte antigen and chemokine receptor 4 (CCR4), which enhances the skin homing ability of VZV-infected T cells and promotes VZV dissemination to the cutaneous site of replication (14). Moreover, VZV infection inhibits the IFN-γ production of VZV-specific CD8^+^ T cells by upregulating the expression of immunoinhibitory proteins programmed death-1 (PD-1) and PD-L1 (15). Along with these phenotypic alterations, VZV-infected T cells show a loss of CD3ϵ expression, leading to TCR-CD3 complex dysregulation and destruction of immune function (16). Fortunately, VZV must infect each T cell individually and cannot induce the fusion of infected T cells (10). The non-infected T cell-triggered immune response is essential for virus clearance and recovery from HZ. In detail, the proportion of CD4^+^ T cells from PBMCs negatively correlated, while regulatory T cells (Tregs) positively correlated with the severity of HZ (11, 17). Besides VZV-specific CD4^+^ T cells are more abundant in PBMCs than CD8^+^ T cells (18, 19). Moreover, the frequency of VZV-specific CD4^+^ T cells peaks at about 2 weeks after HZ onset, then decreases at 3–6 weeks and remains stable for many years (20). Recently, dense CD4^+^ and CD8^+^ T cell infiltrates in ganglia have been observed during HZ (21, 22). Evidence also confirm the key roles of the immune system in the development of PHN, albeit with limited information. A significant decrease of CD4^+^ T cells, CD8^+^ T cells, as well as CD4/CD8 T cell ratio, while an increased percentage of Tregs are validated in patients with PHN compared with those in non-PHN and normal controls, suggesting the more severely impaired T cell-mediated immunity in patients with PHN (11, 23). Thus, a deeper understanding of the immune response of HZ and PHN warrants further investigation to develop potential targets for new immunotherapies.

**Figure 1:**
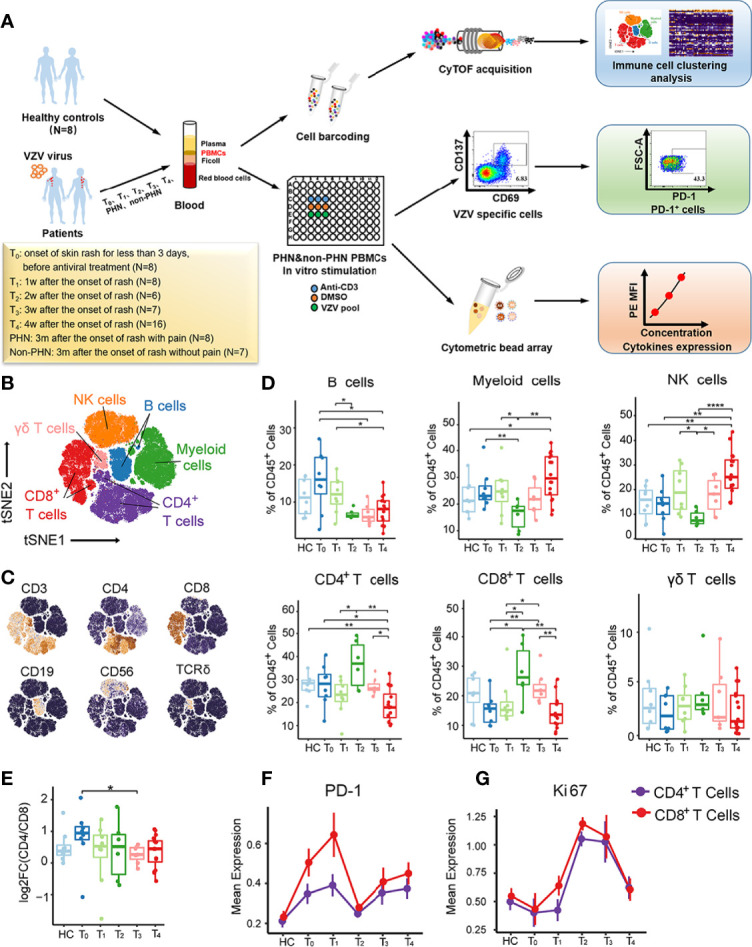
Immune cell profiles of PBMCs from patients with HZ revealed by CyTOF. **(A)** Experimental design of this project. **(B)** t-SNE plot identifying the six major immune cell populations from PBMCs, including CD4^+^ T cells, CD8^+^ T cells, γδ T cells, NK cells, B cells, and myeloid cells. **(C)** t-SNE analysis defining the immune cell populations according to the expression of the main surface markers. **(D)** Boxplots demonstrating the frequencies of the six major immune cells in CD45^+^ cells among healthy controls (HC) and patients with each time point. **(E)** Boxplots showing alteration of CD4/CD8 T cell ratio among HC and patients from T_0_ to T_4_. **(F, G)** The kinetic features of cell markers with PD-1 **(E)** and Ki67 **(F)** in both CD4^+^ T cells and CD8^+^ T cells. *P < 0.05, **P < 0.01, ****P < 0.0001.

To illustrate the immune signatures in PBMCs of patients with HZ and the mechanisms of immune cells in patients with PHN, we utilized the CyTOF to visualize the dynamic immune landscape of PBMCs in patients with HZ and PHN (**Figure 1A**). Our findings depicted two major kinetic signatures of NK and T cell clusters, which closely correlated with clinical pain-related scores. We further demonstrated that patients with PHN had more PD-1^+^CD4^+^, VZV-specific PD-1^+^CD4^+^ T cells, and more TNF-α production than non-PHN patients. Finally, we showed that TNF-α could induce calcium influx in DRG-derived cell lines, suggesting that VZV-specific T cells secreted TNF-α could induce pain. These data illustrated the dynamic signatures of the immune landscape in the peripheral, which closely correlated with clinical features in patients with HZ and revealed the molecular mechanism of VZV-specific T cells induced pain in patients with PHN.

## Materials and Methods

### Patients and Samples

PBMCs for CyTOF analysis were collected from 60 patients at different time points after the onset of rash and eight age-sex-matched healthy donors as controls (HC) (**Supplementary Table 1**, **Figure S1A**). patients with HZ were diagnosed clinically by a typical vesicular rash in dermatome distribution, and medications prescribed were in standard criterion. Exclusion criteria included known serious immunity disorders or malignant disease, serious cardiocerebrovascular or hepatorenal insufficiency, diabetes, hypersensitivity to antiviral or analgesic drugs, and previous use of corticosteroid therapy, and breastfeeding or pregnancy. Clinical pain-related scores, including Numbering Rating Score (NRS), Touch induced NRS, Numbness degree, DN4, ID-pain, GAD7, PHQ9, impact of pain on mood, and impact of pain on daily life were documented in each patient. The clinical characteristics of all the samples were summarized in **Supplementary Table 1**.

We recruited another cohort of 35 patients with five samples at each time point and five HC (**Supplementary Table 2**) to further explore the function of VZV-specific cell subsets. This study was approved by the ethics committee at the First Affiliated Hospital, School of Medicine, Zhejiang University (NO.2018-530), in accordance with the ethical principles of the Declaration of Helsinki. Written informed consent was obtained from all participants before study entry.

### PBMCs Isolation

For PBMCs isolation, whole blood of patients infected with VZV and HC were collected in 10 mL K_2_EDTA coated vacutainer tubes (BD Biosciences). PBMCs were isolated by Ficoll-Paque PLUS (GE Healthcare). PBMCs were washed twice using FACS buffer (0.2% BSA in PBS) at 400 g for 10 min, resuspended, and cryopreserved at a density of 5 × 10^6^ cells for storage.

### CyTOF Samples Processing, Acquisition and Analysis

All the CyTOF data were collected at PLT Company (Hangzhou, China) following their protocols. Briefly, Mass cytometry antibodies are shown in **Supplementary Table 3**. For each sample, 3 × 10^6^ PBMCs were stained with mass-tagged barcodes for 30 min. After washing, every 10 samples were combined and stained with 1μM Cisplatin (Fluidigm) to distinguish live/dead cells at room temperature for 5 min. After washing, cells were incubated with total mouse and human IgG for blocking for 20 min, followed by cell surface markers staining for 30 min on ice. Subsequently, cells were fixed and permeabilized with paraformaldehyde and labeled with DNA Intercalator-Ir overnight, and then incubated with intracellular antibodies. Finally, cells were washed twice with deionized water and diluted with EQ normalization beads containing ^140^Ce, ^151^Eu, ^153^Eu, ^165^Ho, and ^175^Lu (Fluidigm), and the data were acquired by the CyTOF system (Helios, Fluidigm).

A doublet filtering scheme with mass-tagged barcodes was firstly used to debarcode the CyTOF data. Next, the live, singlet, and valid immune cells were obtained *via* manually gating. The data from different batches have been normalized by the bead normalization method. The X-SHIFT (Phenograph) algorithm was used for all samples. CyTOF data was visualized using the t-SNE algorithm, an implemented function in the Rtsne package. The top 1% was deleted, and the 99th was defined as the maximum intensity to exclude extreme marker intensity. The intensity of all markers was then rescaled between 0 and 1. Heatmap of normalized mean expression of markers was generated by R package pheatmap. Spearman correlation was used to explore the relationships between clusters, and clinical traits and visualized using ggplot2.

### Antigen-Specific T Cell Stimulation

Cryopreserved PBMCs were thawed in a complete PRMI 1640 medium containing 10% FBS, 1% penicillin/streptomycin, and 1% L-glutamine at 1 × 10^7^ cells per mL. Three VZV-specific peptide pools (gE, IE62, IE63) (JPT Peptide Technologies, Germany) were mixed together, and PBMCs with 100μL/well were stimulated with 1 μg/mL mixed VZV-specific peptide pools in U-bottom 96-well plate (Jet Bio-Filtration Co., Ltd. China). The same volume of DMSO (Sigma) 1% (vol/vol) was used as a negative Control group and 10 μg/mL purified anti-human CD3ϵ antibody (OKT3, Biolegend) was performed as a positive Control group. After 24 hr stimulation, supernatants were harvested and stored at −80°C for cytokine detection. PBMCs were stained for flow cytometry analysis.

### Flow Cytometry

All the antibodies for flow cytometry were purchased from Biolegend unless otherwise stated. After VZV peptide pools stimulation, the cells were stained with live/dead Zombie violet™ Fixable Viability Kit (Biolegend), Pacific Blue™ anti-human CD56 (clone 5.1H11), Pacific Blue™ anti-human CD14 (clone HCD14), Pacific Blue™ anti-human CD19 (clone HIB19), PE/Dazzle™ 594 anti-human CD3ϵ (clone HIT3a), FITC anti-human CD4 (clone A161A1), Percp/Cyanine5.5 anti-human CD8 (clone SK1), APC anti-human CD137 (clone 4B4-1), PE anti-human CD69 (Clone FN50), and APC/Cyanine7 anti-human PD-1 (clone EH12.2H7). After 30 min incubation, samples were washed twice with FACS buffer, resuspended in 300 μL FACS buffer, and then analyzed on a BD FACSFortessa multicolor flow cytometer (BD Biosciences).

### Cytometric Bead Array

The cytometric bead array (CBA, BD Biosciences) was performed as per the manual instructions. Briefly, the collected supernatants were incubated with human granzyme B (D7 channel), human IL-2 (A4 channel), human IFN-γ (B8 channel), and human TNF-α (C4 channel) beads for 1hr at room temperature in darkness, followed by 50 μL PE detection reagent for another 1hr. Beads were centrifuged, washed, resuspended with wash buffer, and performed on BD FACSFortessa multicolor flow cytometer (BD Biosciences). The data were analyzed using FCAP array software (BD Biosciences).

### ND7/23 Cell Line Culture and Differentiation

DRG neuron-derived ND7/23 cell lines (National Infrastructure of Cell Line Resource, China) were expanded in DMEM medium containing 10% FBS, 1% penicillin/streptomycin, and 1% L-glutamine. The cells were cultured at 1 × 10^6^ cells per mL and passaged every 2 days. When the cells were under logarithmic growth phase, 5000 cells were seeded into a confocal dish (Wuxi NEST Biotechnology Co., Ltd, China) with a medium with 1 mM N6,2’-O-Dibutyryladenosine 3’,5’-cyclic monophosphate sodium salt (cAMP, Sigma-Aldrich) and 10 ng/mL recombinant rat β-nerve growth factor (NGF; R&D Systems Inc.) for differentiation. Cells were maintained in differentiation media for 3 days and then performed a further experiment.

### Measurement of Cytosolic Ca^2+^

As previously described by Ma et al. (24), the calcium imaging method was performed. Briefly, the differentiated ND7/23 cells were washed with FACS buffer containing 2 mM CaCl_2_ for three times and then incubated with 5 μM Fluo-4 AM (Yeasen Biotechnology Co., Ltd, China) for 40 minutes at 37°C. Following this, cells were washed by FACS for another three times and then preserved in 200 μL FACS buffer. Time-lapse images were acquired by Olympus IX83-FV3000-OSR (Olympus Optical Co., Ltd, Tokyo, Japan) with excitation at 488 nm and emission at 500–600 nm. Before the addition of stimulators, seven baseline fluorescence readings were taken, followed by fluorescent readings every second for 300s. The ratio of real-time fluorescence divided by baseline fluorescence (F/F_base_) was utilized for each well to normalize the Ca^2+^ signals.

### Statistical Analysis

All data were expressed as mean ± SEM and analyzed by SPSS 25.0 statistical software (SPSS lnc., Chicago, IL, USA). Data were analyzed by the Kolmogorov-Smirnov test to identify the normal distribution. Non-parametric Wilcoxon test was used for the two groups’ comparison. Spearman’s correlation analysis was used to identify the clinical correlations. A value of *P* < 0.05 was considered statistically significant (**** *P* < 0.0001, *** *P* < 0.001, ** *P* < 0.01, * *P* < 0.05).

## Results

### High-Dimensional Immune Cell Profiling of PBMCs From Patients With HZ During Acute Phase

To comprehensively understand the immunological cell profiles of PBMCs in HZ, we performed CyTOF to analyze PBMC samples from 45 patients and eight age-sex-matched HC (**Figure S1A**). The samples were collected from the designated HZ patient cohort at different time points, including the onset of skin rash for less than 3 days (T_0_, N = 8), 1 week (T_1_, N = 8), 2 weeks (T_2_, N = 6), 3 weeks (T_3_, N = 7), and 4 weeks (T_4_, N = 16) (**Figure 1A**). The detailed sample information and clinical characteristics were listed in **Supplementary Table 1**. To explore the signature of major immune lineages of patients’ PBMCs, we clustered CD45^+^, ProMBP-1^-,^ and CD66b^-^ cells to analyze immune cells without granulocytes. We then characterized six major clusters according to the main immune cell markers, including CD4^+^ T cells, CD8^+^ T cells, γδ T cells, NK cells, B cells and myeloid cells, as displayed *via* t-distributed Stochastic Neighbor Embedding (t-SNE) analysis (**Figures 1B, C**, and **S1B**). It is noteworthy that all these clusters presented their specific kinetics at the different time points (**Figure 1D**). Specifically, B cells showed the highest frequency at T_0_ while decreasing from T_1_ to T_4_, whereas myeloid cells and NK cells reached the lowest percentage at T_2_, then increased at T_3_ and arrived at a peak at T_4_. The frequency of γδ T cells stably maintained at low levels during all the acute phases for T cells. CD4^+^ T cells were firstly upregulated to the peak at T_2_, and then gradually downregulated from T_3_ to T_4_, consistent with the consensus that adaptive immune response usually reaches its maximum two weeks after antigens stimulate T cells. Such a similar kinetic trend was also visualized for CD8^+^ T cells. In addition, a decreased tendency of CD4/CD8 T cell ratio during the acute phases was observed, indicating a faster proliferation velocity of CD8^+^ T cells (**Figure 1E**).

Next, we analyzed the dynamic changes of several vital cellular markers of CD4^+^ and CD8^+^ T cells (**Figures 1F, G**, and **S1C**). The expression of PD-1 on both subsets presented two peaks at T_1_ and T_4_ (**Figure 1F**). For Ki67, only one peak at T_2_ was detected, indicating the strongest proliferation capacity of CD4^+^ and CD8^+^ T cells (**Figure 1G**). This was consistent with their highest frequencies in CD45^+^ cells at T_2_ (**Figure 1D**). Collectivelly, these data emphasize the dynamic fluctuation of six major clusters from PBMCs, especially for CD4^+^ and CD8^+^ T cells.

### Dynamic Characteristics of Different Immune Subsets

**Figure 2:**
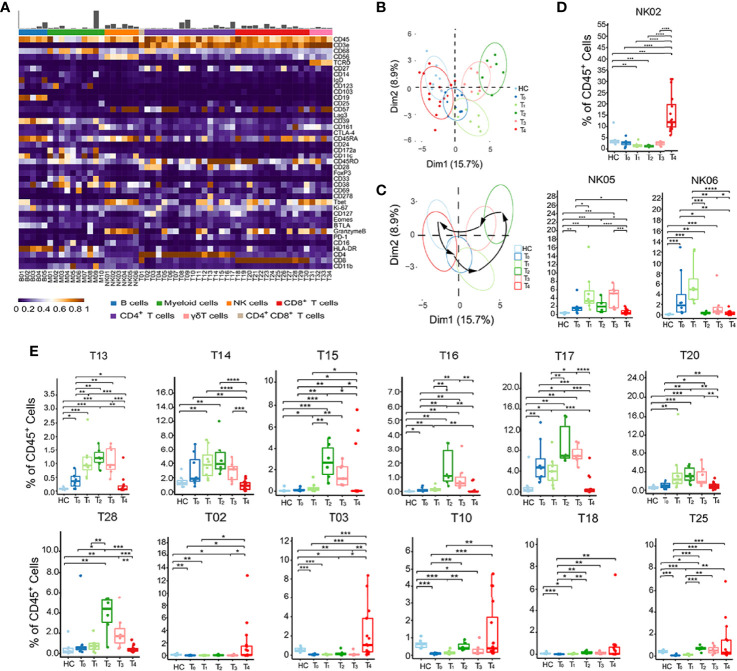
Identification of dynamic signatures of each immune subset. **(A)** Heatmap showing the normalized mean expression of 40 membranous or intracellular markers to identify the phenotypes of the main six immune cell clusters. We characterized five B cell clusters (B01–B05), 10 myeloid cell clusters (M01–M10), six NK cell clusters (NK01–NK06), 16 CD4^+^ T cell clusters (T02–T17), 13 CD8^+^ T cell clusters (T18–T30), four γδ T cell clusters (T31–T34). Relative frequency was shown as a bar graph on the top. **(B, C)** Principal component analysis (PCA) showing **(B)** the distinct immune signatures among HC and patients with each time point from T_0_ to T_4_. **(C)** Black arrows indicate the rhythmic changes with the disease development. **(D, E)** Boxplots revealing the dynamic characteristics and frequencies of different immune cell clusters, NK cells shown in **(D)** and T cells shown in **(E)**. *P < 0.05, **P < 0.01, ***P < 0.001, **** P < 0.0001.

To further characterize the detailed phenotypes of these six major clusters, we analyzed immune cell clusters with 40 cell markers by algorithm X-shift and visualized them in a heatmap (**Figure 2A**). In general, we identified 5 B cell subsets (B01–B05), 10 myeloid cell subsets (M01–M10), 6 NK cell subsets (NK01–NK06), 16 CD4^+^ T cell subsets (T02–T17), 13 CD8^+^ T cell subsets (T18–T30), and 4 γδ T cell subsets (T31–T34) (**Figure 2A**, **Supplementary Table 4**). Using principal component analysis (PCA), we revealed that the signature of immune cells at different time points was distinct and manifested a rhythmic change (**Figures 2B, C**). Compared to that in HC, the immune signature of patients gradually increased from T_0_ to T_2_, but progressively went back from T_3_ to T_4_, showing a good correlation between the dynamic immune signature and the disease development during acute phases (**Figure 2C**).

Next, we performed the frequencies of all immune cell subsets at each time point from T_0_ to T_4_ to investigate the specific dynamic features of each phenotype (**Figures 2D, E**, and **S2A**). Among the 6 NK cell subsets, NK02 (CD56^low^CD16^+^) frequency only increased at T_4_. In contrast, NK05 and NK06 (CD56^high^CD16^-^) frequencies reached the peak at T_1_ (**Figure 2D**). This phenomenon suggested that the fundamental functions of distinct NK cell subsets varied at different stages. There were also two major dynamic patterns in the case of T cell subsets: quick response pattern and slow response pattern (**Figure 2E**). In a quick response pattern, T cell subsets, such as T13 (CD25^+^FOXP3^+^CD45RO^+^CD4^+^), T14 (CD27^l^°^w^CD45RO^+^CD4^+^), T15, and T16 (GranzymeB^+^Tbet^+^CD45RO^+^CD4^+^), T17 (CD161^+^CD45RO^+^CD4^+^), T20 (PD-1^+^CD45RO^+^CD8^+^) and T28 (CD161^+^CD45RO^+^CD8^+^), responded quickly and reached the highest frequencies at T_2_, and then gradually decreased at T_3_ and T_4_, which was similar to the trend of total CD4^+^ and CD8^+^ T cells (**Figure 1D**). In slow response pattern, the frequencies of T cell subsets, such as T02 (BTLA^+^CD27^+^CD127^+^CD57^l^°^w^CD4^+^), T03 (CD127^high^CD45RO^+^CD4^+^), T10 (CD28^+^CD45RO^+^CD4^+^), T18 (BTLA^+^CD27^+^CD127^+^CD57^l^°^w^CD8^+^), and T25 (CD27^l^°^w^CD45RO^l^°^w^CD8^+^), stably maintained at low levels from T_0_ to T_3_, and only increased at T_4_ (**Figure 2E**). Together, these results suggest two major dynamic signatures in NK, CD4^+^ and CD8^+^ T subsets in patients with HZ.

### Correlations Between Immune Subsets and Clinical Pain-Related Scores

**Figure 3:**
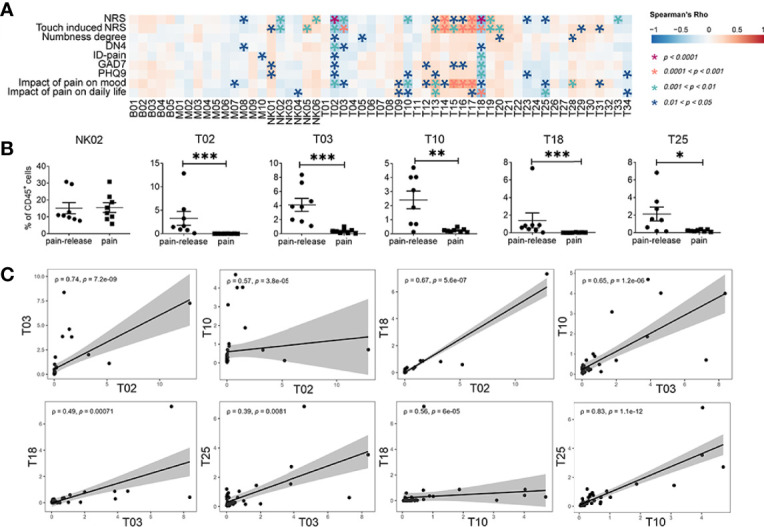
Correlations between immune subsets and clinical pain related scores. **(A)** Spearman’s correlation analysis showing the correlations between each immune cluster and clinical pain-related scores. **(B)** The difference in the frequency of different immune subsets with slow response patterns that negatively correlated with clinical pain-related scores (**Figure 2E**) in the pain or the pain-release groups at the T_4_ time point_._ **(C)** The correlation analysis between each immune subset with slow response patterns. *P < 0.05, **P < 0.01, ***P < 0.001.

Clinical pain-related scores, including NRS, touch induced NRS, numbness degree, DN4 (Douleur Neuropathique 4 Questions), ID-pain, generalized anxiety disorder (GAD7), patient health questionnaire-9 (PHQ9), the impact of pain on mood, and impact of pain on daily life are important parameters to describe the pain levels of patients with HZ. However, these clinical pain-related scores are highly subjective and lack objective clinical indicators. We wondered whether the immune characteristics in PBMCs could be considered objective biomarkers for pain description and potential targets for future immunotherapy for pain-release. Thus, we analyzed the correlation between clinical pain-related scores and immune subsets by Spearman’s correlation (**Figure 3A**). Prior to the correlation analysis, we validated that the clinical parameters significantly increased in patients compared with that in HC, but there was no difference among different time points (**Figure S3A**). This observation hinted the practicability of combining the data at all-time points for clinical parameter-related analysis. B cells and myeloid cells displayed little association with clinical pain-related scores among all the immune clusters. For NK cells, the CD56^high^CD16^-^ NK subset (NK05, NK06) positively correlated with clinical pain-related scores, while the CD56^l^°^w^CD16^+^ NK subset (NK02) presented a negative correlation (**Figure 3A**).

Similar to NK cells, the correlation between clinical pain-related scores and different T cell subsets was also confirmed. Interestingly, we revealed that most T cell subsets with quick response patterns, such as T13, T14, T15, T16, T17, T20, and T28, positively correlated with clinical pain-related scores. However, all T cell subsets with slow response patterns, such as T02, T03, T10, T18, and T25, showed a negative correlation with clinical pain-related scores (**Figures 2E**, **3A**). In addition, at one month after the onset of rash (T_4_ time point), some patients still suffered persistent pain, but the others felt relieved (**Supplementary Table 1**). As the frequencies of immune subsets with slow response patterns only increased at T_4_, we divided patients at T_4_ into pain group and pain-release group and examined the difference between all NK and T cell subsets with slow response patterns in these two groups. We found that T02, T03, T10, T18, and T25 subsets significantly increased in the pain-release group (**Figure 3B**).

We further analyzed the correlation between each immune subset (**Figure S3B**). T cell subsets with quick response patterns (T13-T17, T28-T32) were positively correlated. Similarly, the slow response pattern of T cell subsets between T02 and T03, T10, T18, subsets between T03 and T10, T18, T25, subsets between T10 and T18, T25 also showed positive correlations (**Figures 3C**, **S3B**). T02 (BTLA^+^CD27^+^CD127^+^ CD57^l^°^w^CD4^+^) and T18 (BTLA^+^CD27^+^CD127^+^CD57^l^°^w^CD8^+^) were negatively correlated with most of the clinical pain-related scores and positively correlated with each other, indicating the involvement of BTLA^+^CD27^+^CD127^+^CD57^l^°^w^ T cells in the pathogenesis patients with HZ (**Figures 2A**, **3A, C**). Further investigations are required to explore the functions of BTLA^+^CD27^+^CD127^+^CD57^l^°^w^ T cells in the progression of the acute phase of HZ.

### Comparison of the Immune Landscape Between PHN and Non-PHN Patients

**Figure 4:**
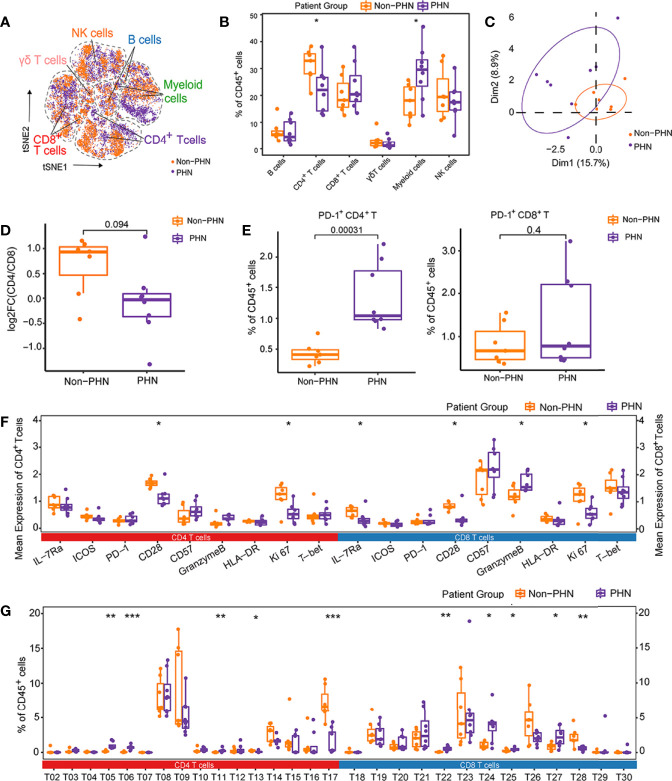
Characteristics of immune landscape of patients with PHN and without PHN. **(A)** t-SNE plots identifying the difference of six main immune cell profiles, as shown in **Figure 1A**, between PHN and non-PHN. **(B)** Boxplots showing the frequencies of indicated clusters in the PHN and non-PHN groups. **(C)** The PCA projections of immune signatures between the PHN and non-PHN groups. **(D)** Boxplots showing the tendency of CD4/CD8 T cells ratio in the PHN group compared with the non-PHN group. **(E)** Boxplots showing the frequencies of PD-1^+^CD4^+^ T cells and PD-1^+^CD8^+^ T cells in PHN and non-PHN groups. **(F)** Boxplots displaying the mean expression levels of important cellular markers on CD4^+^ T cells and CD8^+^ T cells in the PHN and non-PHN groups. **(G)** Boxplots exhibiting the frequencies of T cell clusters in the PHN and non-PHN groups. *P < 0.05, **P < 0.01, ***P < 0.001.

A new cohort was set up with 8 PHN samples and 7 non-PHN samples (**Figure 1A**, **Supplementary Table 1**) to investigate the immune signature in PBMCs between non-PHN and PHN groups by CyTOF analysis. The distribution among non-PHN subjects is similar, and so it is in PHN subjects (**Figure S4A**). However, the distribution of different immune cells varied between non-PHN and PHN groups in t-SNE projections (**Figure 4A**). Compared with the non-PHN group, the percentage of CD4^+^ T cells in the PHN group was significantly decreased, accompanied by a significant increase in myeloid cells (**Figure 4B**). PCA analysis further confirmed the distinct immune signature between the non-PHN and PHN groups (**Figure 4C**). Previous studies showed that patients with PHN had a lower CD4/CD8 T cell ratio than non-PHN patients (11, 23). We observed a similar decrease in CD4/CD8 T cell ratio in PHN but with no significant difference, which the insufficient sample size might cause in each group (**Figure 4D**). Since PD-1 expression was upregulated at the late acute stage of HZ and was indicated as an exhaustion marker (**Figure 1F**). We wondered whether the percentage of PD-1^+^ T cells in peripheral blood differed between PHN and non-PHN group. We found that the frequencies of PD-1^+^CD4^+^ T cells, but not PD-1^+^CD8^+^ T cells, significantly increased in PHN group (**Figure 4E**).

Next, we analyzed the mean expression level of distinct cellular markers on the total CD4^+^ and CD8^+^ T cells between the PHN and non-PHN groups. In PHN group, the markers that are related to T cell activation and proliferation (e.g., CD28, Ki67, and IL-7Ra) were significantly declined in both CD4^+^ and CD8^+^ T cells compared with that in non-PHN group, suggesting the inefficiency of CD4^+^ T cells and CD8^+^ T cells on VZV clearance in patients with PHN (**Figure 4F**). Besides these cellular markers, we also assessed frequencies of different immune cell subsets between the PHN and non-PHN groups (**Figures 4G**, **S4B**). For T cells, compared to the non-PHN group, T13, T17 and T28 subsets were significantly decreased, whereas T05, T06, T11, T22, T24, T25, and T27 subsets were significantly increased in the PHN group (**Figure 4G**). Interestingly, both T17 and T28 were CD161^+^, indicating the involvement of CD161^+^ T cells in PHN development (**Figure 2A**). Among other immune cells, M01 and NK05 clusters dramatically decreased in the PHN group (**Figure S4A**). These data might indicate that the signature of αβ T subsets dominated the difference between the PHN and non-PHN groups. Collectively, these data reveal that many T cell subsets of the PHN group are much different from that of the non-PHN group, which might ultimately be one of the reasons for the initiation and progression of PHN.

### The Different Characteristics of VZV-Specific T Cells Between PHN and Non-PHN Patients

**Figure 5:**
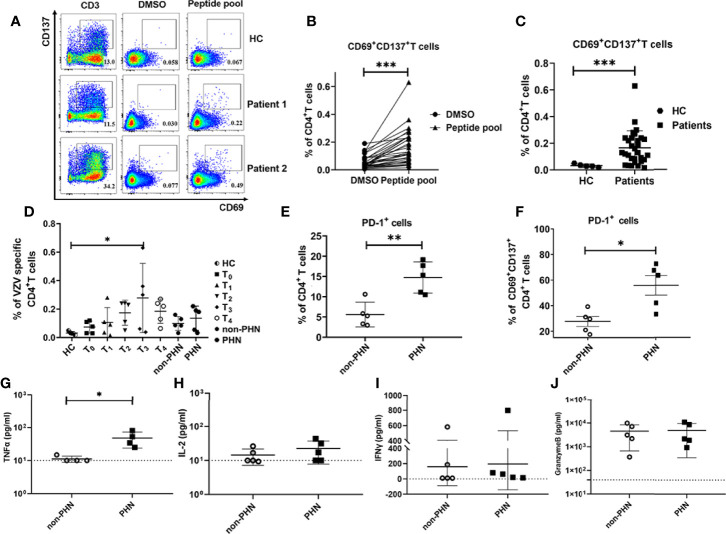
Characteristics of VZV-specific CD4^+^ T cell response. VZV-specific CD4^+^ T cells assessed as frequencies of CD69^+^CD137^+^CD4^+^ T cells after stimulation of PBMCs by VZV peptide pools (gE, IE62, IE63). **(A)** Examples displaying the Fluorescence-activated cell sorting (FACS) plot, gated on total CD4^+^ T cells. The anti-human CD3 antibody group and DMSO group were performed as positive control and negative control, respectively. **(B)** Comparison of the frequency of VZV-specific CD4^+^ T cells in peptide pools or DMSO stimulation. **(C)** Comparison of the frequency of VZV-specific CD4^+^ T cells in patients and HC. **(D)** The characteristics of dynamics of VZV-specific CD4^+^ T cells in patients at different time points after the onset of rash. **(E, F)** Frequencies of PD-1^+^ cells in total CD4^+^ T cells **(E)** and VZV-specific CD4^+^ T cells **(F)** in PHN and non-PHN groups. **(G-J)** Different levels of cytokines in the cultured supernatants of PBMCs with VZV peptide pools stimulation in PHN and non-PHN groups. **(G)**TNF-α, **(H)** IL-2, **(I)** IFN-γ, **(J)** Granzyme B. *P < 0.05, **P < 0.01, ***P < 0.001.

T cells that are activated by TCR signals can express multiple activation markers, including CD69, CD137, CD154, and OX40, which are used to identify pathogen-specific T cells (25, 26). We used VZV peptide pools to stimulate PBMCs from patients with HZ at different time points after the onset of rash to elucidate the role of VZV-specific T cells play in patients with HZ. VZV-specific T cells were referred as CD69^+^CD137^+^, and the gating strategies were displayed in **Figure S5A**. For PBMCs stimulation, three VZV peptide pools (gE, IE62, IE63) were mixed as stimulators, and the anti-human CD3ϵ and DMSO were performed as positive and negative controls, respectively (**Figures 5A**, **S5B**). We identified VZV-specific CD4^+^ and CD8^+^ T cells in all patients with HZ using VZV peptide pools compared to DMSO control (**Figures 5B**, **S5C**). Compared with HC, the VZV-specific CD4^+^ T cells were significantly elevated, which is important for virus clearance (**Figure 5C**). However, no significant increase was found in VZV-specific CD8^+^ T cells (**Figure S5D**). We revealed the highest frequency of VZV-specific CD4^+^ T cells at T_3_, in accordance with the expression of Ki67 at T_3_, suggesting the strongest antiviral response during this time point (**Figures 1F**, **5D**). Although there was no difference for VZV-specific CD8^+^ T cells, a similar increased tendency was also detected at T_3_ (**Figure S5E**). Interestingly, there was no difference between the frequencies of both VZV-specific CD4^+^ and CD8^+^ T cells in the PHN and non-PHN groups (**Figures 5D**, **S5E**).

Since a remarkable increase of PD-1^+^CD4^+^ T cells in patients with PHN has been identified as previously described (**Figure 4E**), we compared the frequencies of PD-1^+^ T cells in total CD4^+^ T cells and VZV-specific CD4^+^ T cells in non-PHN and PHN groups. Consistent with our CyTOF data, the frequency of both total PD-1^+^CD4^+^ T cells and VZV-specific PD-1^+^CD4^+^ T cells in the PHN group was higher than that in the non-PHN group (**Figures 5E, F**). In terms of CD8^+^ T cells, the frequencies of total PD-1^+^CD8^+^ T cells and PD-1^+^ VZV-specific CD8^+^ T cells were similar in the two groups (**Figures S5F, G**).

To evaluate the function and response of VZV-specific T cells, we further explored the cytokine secretion of PBMCs in the supernatant after stimulation with VZV peptide pools. Among the four cytokines, including TNF-α, IL-2, IFN-γ, and granzyme B, the production of TNF-α was significantly higher in the PHN group than that in the non-PHN group, suggesting the positive effect of TNF-α on PHN progression (**Figures 5G–J**).

**Figure 6:**
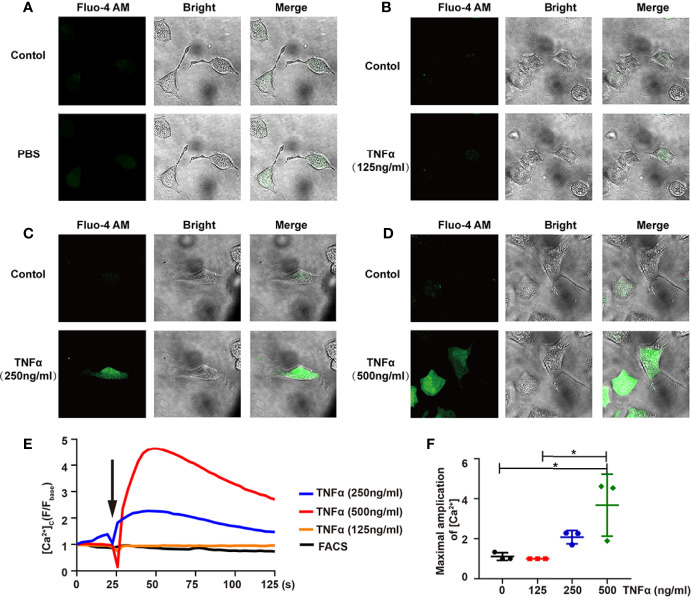
TNF-α induces Ca^2+^ influx in differentiated ND7/23 cells. **(A-D)** The relative intensity of Fluo-4 AM fluorescence - and bright-field images were shown in PBS **(A)**, 125ng/mL TNF-α **(B)**, 250ng/mL TNF-α **(C)**, 500ng/mL TNF-α **(D)**. The Control group indicated the background before adding the stimulator. Images were taken by confocal microscope. **(E)** Confocal microscope analysis of [Ca^2+^] in ND7/23 cells with PBS and TNF-α stimulation. Arrow indicated cells stimulated by PBS or TNF-α. **(F)** Quantitative analysis of the maximal increased level of cytosolic Ca^2+^ after stimulations. *P < 0.05.

TNF-α played an important role in neuropathic pain (27). Calcium influx was one of the indicators for nociceptive responses, such as pain, in dorsal root ganglion (DRG) (28). Since there is no PHN mouse model (29), we visualized the dynamic of Ca^2+^ influx in DRG neuron-derived ND7/23 cells *via* TNF-α stimulation. Compared with the PBS group, the Ca2+ intensity in DRG cells was enhanced after TNF-α stimulation (**Figures 6A–D**), and, more importantly, in a TNF-α dose-dependent manner (**Figure 6E**, **Supplementary video 1** **–** **4**). The maximal increased level of Ca^2+^ by 500ng/mL TNF-α stimulation was significantly higher than that by 125ng/mL TNF-α stimulation (**Figure 6F**).

Collectively, our data indicated that VZV-specific CD4^+^ T cells, but not CD8^+^ T cells, dramatically changed in acute stages and were more exhausted in the PHN group. In addition, the observation of higher expression of TNF-α after VZV peptide pool stimulation in PHN than that in non-PHN patients and the subsequent effect on Ca^2+^ influx in DRG-derived cells implies that VZV-specific T cells play a key role in the progression of pain in patients with PHN.

## Discussion

In this study, we performed a CyTOF analysis of PBMCs of patients with PHN and HZ in the long-term cohort at six-time points after HZ onset. We further explored the characteristics of VZV-specific T cells by VZV peptide pools stimulation. These data promoted a comprehensive understanding of the immune cell profiles and kinetics and the role of VZV-specific T cells in PHN pathogenesis.

NK cells are important sentinels of the immune system, which function as front-line responders and warn the host of infections. During the early phase of acute inflammation, secondary to an infection, NK cells mainly present regulatory abilities *via* diverse cytokine production upon activation (30). Moreover, the highly proliferative CD56^high^CD16^-^ NK cell subset mainly participates in immunomodulatory cytokine production, including IFN-γ, TNF-β, IL-10, IL-13, and GM-CSF (31). We observed that CD56^high^CD16^-^ NK cells were the predominant subset of NK cells at T_1_ in line with these results. Compared with the CD16^-^ NK subset, expression of CD16 on NK cells renders strong mediators of antibody-dependent cellular cytotoxicity against IgG coated target cells, because the combination of CD16 and its ligand IgG Fc induces the polarization and degranulation of NK cells (32). The CD56^l^°^w^CD16^+^ subset, accounting for the majority (~90%), is potently cytotoxic, albeit lowly proliferative (31). In our study, the percentages of the CD56^l^°^w^CD16^+^ subset increased to the highest level at T_4_, indicating the potent cytotoxicity of eliminating VZV-infected cells. We also found that the CD56^high^CD16^-^ NK subset positively correlated with pain-related scores, while the CD56^l^°^w^CD16^+^ NK subset presented a negative correlation. This suggested that the higher frequency of CD56^high^CD16^-^ NK cells during the early acute phase indicated the severe VZV infection, and the increasing frequency of CD56^low^CD16^+^ NK cells during the late acute phase signified the stronger capacity to obliterate VZV infection.

During HZ, total CD4^+^ and CD8^+^ T cells simultaneously reached the highest frequencies with the highest expression of Ki67 at T_2_, indicating the strongest antiviral response and proliferation ability of these cells. In addition, a similar trend was observed in T cell subsets with quick response patterns, including T13, T14, T15, T16, T17, T20, and T28. These subsets were memory T cells with CD45RO^+^ and positively correlated with the pain-related scores. T13, the Tregs (CD25^+^FOXP3^+^CD45RO^+^CD4^+^), is essential to maintain immune homeostasis *via* governing aggravated and destructive inflammation, exhibiting protective roles in the host during viral infections (33). Investigations have revealed their suppressive roles in controlling the antiviral CD4^+^ and CD8^+^ T cells in chronic Hepatitis B/C, and HSV-1 infection (34–36). Due to the immune suppression of Tregs, effector T cell-mediated antiviral immune responses are inhibited, thus facilitating viral persistence and disease exacerbation (37).

In contrast to Tregs, T15, and T16 (Tbet^+^CD45RO^+^CD4^+^), the typical Th1 effector subsets, mediate immune response against intracellular pathogens and rapidly produce IFN-γ, IL-2, and TNF-α to help control acute infection (38). The higher frequency of these two subsets suggested the severe VZV infection in the early acute HZ phase, leading to the pain-related syndromes. The subsets of T17 and T28 are represented as CD161^+^CD4^+^ and CD161^+^CD8^+^ T cells. It is reported that T cells subsets capable of producing IL-17 are virtually restricted to express CD161 (39, 40). The positive correlation between T17 or T28 and pain-related scores indicated that IL-17 was positively involved in the host immune response related to pain. The T20 (PD-1^+^CD8^+^) subset presented a transient induction of PD-1 expression on CD8^+^ T cells in the acute phase. Previous studies have documented that this transient PD-1 upregulation has little influence on early CD8^+^ T cell activation, expansion, and effector differentiation during acute viral infection (41). Moreover, the transient PD-1^+^CD8^+^ T cells can exert similar effector molecules, including granzyme B, IFN-γ, TNF-α (41). Coincidentally, the T20 subset was granzyme B positive in our data, which indicated the active antiviral role of T20 in the acute HZ phase.

Compared with the cell subsets with quick response patterns, we noted that several cell subsets with slow response patterns only increased at T_4_, such as T02, T03, T10, T18, and T25. In addition, all of these subsets negatively correlated with the pain-related scores and showed increased frequencies in the pain-release group at T_4_. Among these subsets, T02 (BTLA^+^CD27^+^CD127^+^CD57^l^°^w^CD4^+^) and T18 (BTLA^+^CD27^+^CD127^+^CD57^l^°^w^CD8^+^) subsets showed a significantly positive correlation between each other. BTLA, also known as B and T lymphocyte attenuator, is a co-inhibitory receptor of the CD28 superfamily, which plays an important role in T cell functions *via* binding with its ligand herpesvirus entry mediator (HVEM) (42). Many studies have documented the negative modulation on T cell activation and proliferation in various infectious diseases, such as COVID-19, HBV, and cytomegalovirus (43). In contrast, BTLA can also function as an activating ligand. BTLA expression on αβ T cells is essential for cells to exhibit an active central memory phenotype against *M. tuberculosis* (Mtb) infection with a strong ability to produce IFN-γ and perforin (44). In addition, the BTLA-HVEM combination in a *cis*-heterodimeric complex can inhibit the activation of the HVEM-dependent NF-κB signaling pathway, and then helps maintain T cells in a naïve state (45). In our study, the negative correlation of BTLA^+^ αβ T cells with clinical pain-related scores and the significantly elevated frequencies in the pain-release group at T_4_ indicated the similarly protective immune response of BTLA^+^ αβ T cells against VZV infection. Further investigation will be performed to validate this assumption.

T cell exhaustion is reported as a common appearance during persisting infections, such as chronic lymphocytic choriomeningitis virus (LCMV) and HIV (46, 47). The exhausted T cells are characterized by impaired effector functions, sustained expression of multiple inhibitory receptors, especially the typical cellular marker PD-1, and a transcriptional signature that is different from that of functional effector or memory T cells (48). We found a slight increase in PD-1 expression on both CD4^+^ and CD8^+^ T cells from T2 to T4 during the acute phase of HZ. In addition, frequencies of PD-1^+^CD4^+^ T cells in patients with PHN were significantly higher than that in non-PHN patients. The data implied that the severity of the disease, at least partly, correlated with the extent of T cell exhaustion. By stimulating PBMCs with the VZV peptide pools, the expression of PD-1 on CD69^+^CD137^+^CD4^+^ T cells was significantly higher in the PHN group than that in the non-PHN group, but the frequency of virus-specific CD8^+^ T cells was similar in two groups, indicating that virus-specific CD4^+^ T cells were more exhausted in the PHN group than in the non-PHN group. It has been demonstrated that the blocking axis of PD-1 and PD-1 ligand could reinvigorate virus-specific T cell responses and lead to a lower viral load during chronic LCMV infection (49). Therefore, PD-1 would be a potential target for reversing the dysfunction of exhausted VZV-specific CD4^+^ T cells to improve the control of VZV infection.

Strikingly, we also identified a significant elevation of TNF-α in the supernatant of PBMCs by VZV peptide pools stimulation in the PHN group. It has been reported that TNF-α is essential for anti-tumoral but not for antiviral response in T cells (50). Therefore, the elevated level of TNF-α in patients with PHN may not benefit VZV clearance but rather pain persistence in patients with PHN. Indeed, TNF-α affected the Ca^2+^ influx of DRG in a dose-dependent manner. These data suggested the active roles of TNF-α in promoting pain symptoms. Wagner et al. has first validated that TNF-α, a well-known immune and pro-inflammatory mediator, exhibits a similar hyperalgesia after the injection into the nerve in 1996 (51). Since then, increasing evidence has put forward the roles of TNF-α in the mechanisms of both peripheral and central neuropathic pain (52, 53). However, we noticed that the concentration of TNF-α in the supernatant after VZV peptide pool stimulation was ~10000 times lower than the concentration of TNF-α for Ca^2+^ influx of DRG-derived cells (**Figures 4G**, **6F**). This difference may be because experiments *in vitro* could not truly reflect situations *in vivo*. We hypothesized that the local concentration of TNF-α surrounding VZV-specific T cells in ganglia was high enough to stimulate DRG neurons. In fact, by analyzing ganglia in cadavers with active shingles before death, the Abendroth group has found that both CD4^+^ and CD8^+^ T cells could infiltrate into ganglia and secrete granzyme B (22). Therefore, it is possible that during HZ, the infection of VZV induces T cell-mediated immune response, then these VZV-specific T cells infiltrate into ganglia, stimulated by VZV antigens, and secrete TNF-α to induce pain in patients with PHN. This hypothesis requires to be examined by establishing a new PHN animal model. Regardless, we found TNF-α produced by VZV-specific T cells could be one of the reasons for persistent pain in patients with PHN, and the effective blockade of TNF-α would be a promising treatment in the mediation of pain induced by VZV infection.

In conclusion, we have comprehensively shown the dynamic immune landscape of patients with HZ, and documented two major dynamic features and their close correlation with clinical pain-related scores. We also revealed the enhanced frequencies of total PD-1^+^CD4^+^, VZV-specific PD-1^+^CD4^+^ T cells, and TNF-α content in the PHN group. Moreover, TNF-α could induce the Ca^2+^ influx of DRG-derived cells in a dose-dependent manner. Based on these results, we consider that the TNF-α secreted by VZV-specific T cells that function on DRG then induce pain. These findings provide a better understanding of VZV pathogenesis and immunity mechanisms, offering a fundamental basis for exploring new therapies for VZV infection.

## Data Availability Statement

The original contributions presented in the study are included in the article/**Supplementary Material**. Further inquiries can be directed to the corresponding authors.

## Ethics Statement

The studies involving human participants were reviewed and approved by the First Affiliated Hospital, School of Medicine, Zhejiang University (NO.2018-530). The patients/participants provided their written informed consent to participate in this study. Written informed consent was obtained from the individual(s) for the publication of any potentially identifiable images or data included in this article.

## Author Contributions

XZ and ZF designed and supervised the project. QP performed the experiments. XG collected the patient samples and clinical characteristics. YL (3rd author) analyzed the CyTOF data. QP, GW and LZ processed the samples. QP and XZ wrote the manuscript and revised the manuscript. All authors contributed to the article and approved the submitted version.

## Funding

This work was supported by Zhejiang Provincial Department of Science and Technology (2022C03081 to ZF), the National Natural Science Foundation of China (31870899, 32070899 to XZ, and 82103304 to QP) and the Independent Task of State Key Laboratory for Diagnosis and Treatment of Infectious Diseases (2022zz07 to QP).

## Conflict of Interest

A patent application has been submitted based in part on results presented in this manuscript. QP, XG, XZ and ZF are listed as the inventors.

The remaining authors declare that the research was conducted in the absence of any commercial or financial relationships that could be construed as a potential conflict of interest.

## Publisher’s Note

All claims expressed in this article are solely those of the authors and do not necessarily represent those of their affiliated organizations, or those of the publisher, the editors and the reviewers. Any product that may be evaluated in this article, or claim that may be made by its manufacturer, is not guaranteed or endorsed by the publisher.

## Supplementary Material

Supplementary Figure 1 Overview of markers expression by CyTOF data analysis (related to **Figure 1**). **(A)** The comparison of age and sex between patients and HC for CyTOF analysis. **(B)** The t-SNE plots of selected markers as shown in **Figure 1B**. **(C)** The dynamic features of mean expression level of cell markers in CD4^+^ and CD8^+^ T cells.

Supplementary Figure 2 Dynamic signatures of immune subsets in HZ patients at different time point after the onset of rash (related to **Figure 2**). **(A)** Boxplots revealing the dynamic characteristics of immune subsets as shown in .

Supplementary Figure 3 The clinical pain-related scores at different time point and correlation analysis between each immune cluster in HZ patients (related to **Figure 3**). **(A)** Comparisons of clinical pain-related scores, including NRS, Touch induced NRS, Numbness degree, DN4, ID-pain, GAD7, PHQ-9, Impact of pain on mood, and Impact of pain on daily life among HC and patients from T_0_ to T_4_. **(B)** The correlation analysis among immune subsets identified by CyTOF. The red boxes indicate positive correlation and blue boxes indicate negative correlation.

Supplementary Figure 4 The immune landscape of PBMCs in PHN and non-PHN patients (related to **Figure 4**). **(A)** the merged tSNE plot of each PHN and non-PHN sample. **(B)** Boxplots exhibiting the frequencies of different phenotypes of B cells, myeloid cells, NK cells, and γδ T cells in PHN and non-PHN groups.

Supplementary Figure 5 The characteristics of VZV specific CD8^+^ T cell response (related to **Figure 5**). **(A)** The gating strategy for getting CD69^+^CD137^+^CD4^+^ T cells or CD69^+^CD137^+^CD8^+^ T cells. Dead cells and pacific blue positive cells are excluded for analysis (details in method). **(B)** Examples displaying the FACS plot, gated on total CD8^+^ T cells. **(C)** Comparison of frequency of VZV specific CD8^+^ T cells in peptide pools or DMSO. **(D)** Comparison of frequency of VZV specific CD8^+^ T cells in patients and HC. **(E)** The characteristics of dynamics of VZV-specific CD8^+^ T cells in patients at different time point after the onset of rash. **(F, G)** Percentages of PD-1^+^ cells in total CD8^+^ T cells **(F)** and VZV specific CD8^+^ T cells **(G)** at different time point after the onset of rash.

Supplementary Video 1–4 (related to **Figure 6**). The changes of Fluo-4 AM signal intensity of ND7/23 cell line with the stimulation by PBS (Video1) and 125ng/mL TNF-α (Video2), 250ng/mL TNF-α (Video3), 500ng/mL TNF-α (Video4). The videos were taken by confocal microscope.
