# Supplementary figures and images for: Dynamic Immune Landscape and VZV-Specific T Cell Responses in Patients With Herpes Zoster and Postherpetic Neuralgia
Source: Front Immunol. 2022 Jun 1;13:887892. doi: 10.3389/fimmu.2022.887892 (PMC9199063; doi:10.3389/fimmu.2022.887892)

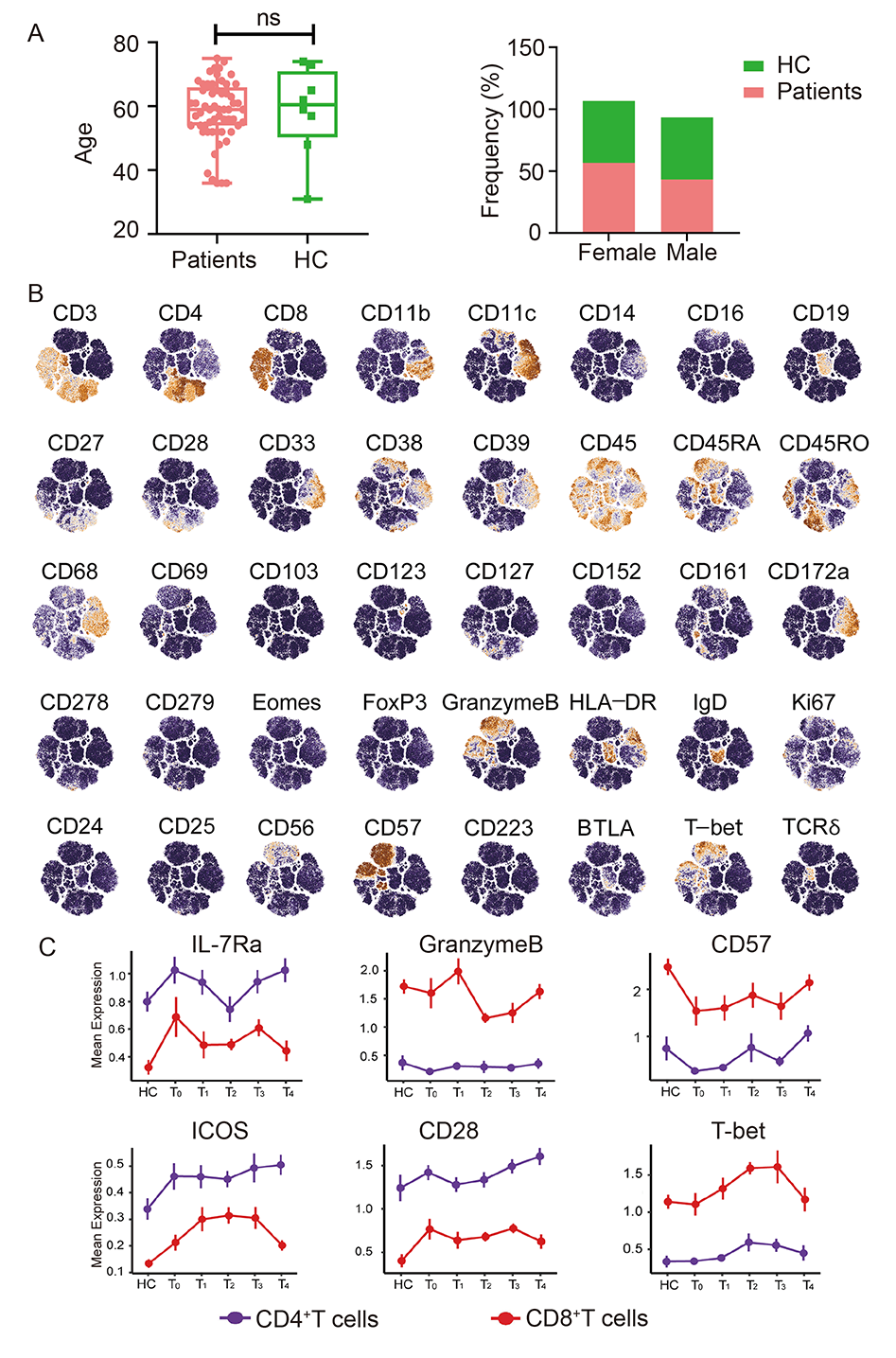

Supplement: Supplementary Figure 1 — Overview of markers expression by CyTOF data analysis (related to Figure 1 ). (A) The comparison of age and sex between patients and HC for CyTOF analysis. (B) The t-SNE plots of selected markers as shown in Figure 1B . (C) The dynamic features of mean expression level of cell markers in CD4+ and CD8+ T cells. [file Image_1.tif]

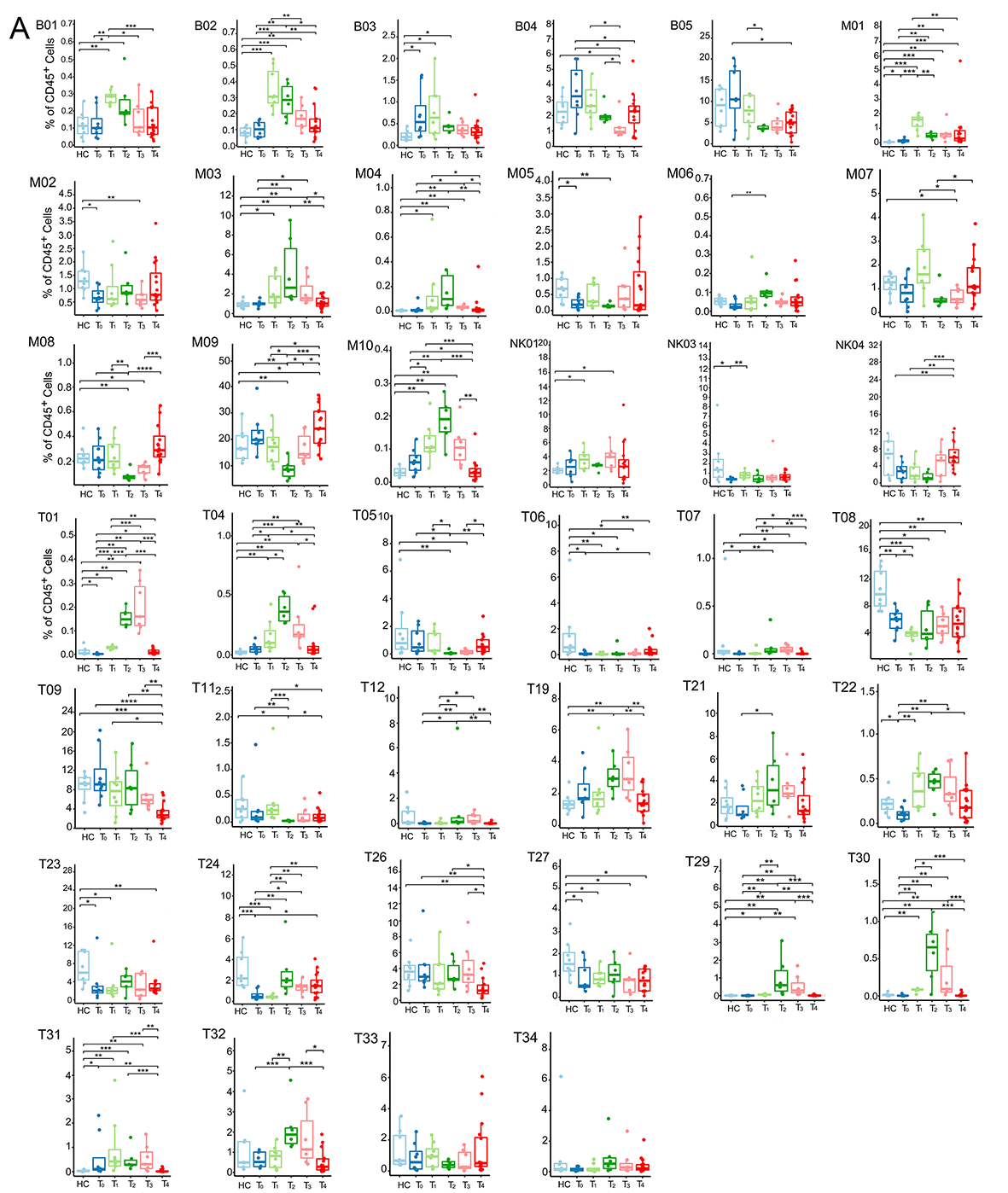

Supplement: Supplementary Figure 2 — Dynamic signatures of immune subsets in HZ patients at different time point after the onset of rash (related to Figure 2 ). (A) Boxplots revealing the dynamic characteristics of immune subsets as shown in . [file Image_2.tif]

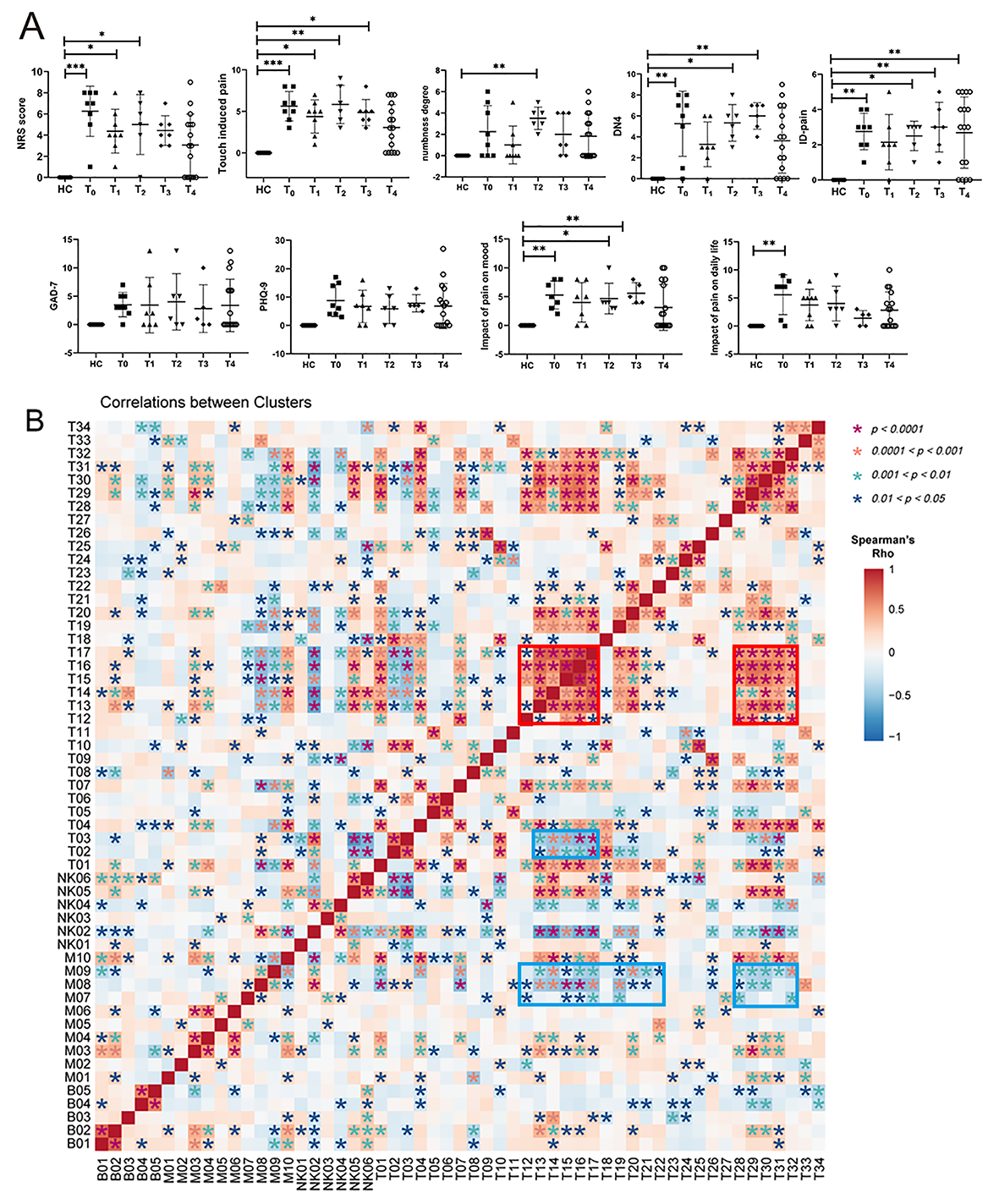

Supplement: Supplementary Figure 3 — The clinical pain-related scores at different time point and correlation analysis between each immune cluster in HZ patients (related to Figure 3 ). (A) Comparisons of clinical pain-related scores, including NRS, Touch induced NRS, Numbness degree, DN4, ID-pain, GAD7, PHQ-9, Impact of pain on mood, and Impact of pain on daily life among HC and patients from T0 to T4. (B) The correlation analysis among immune subsets identified by CyTOF. The red boxes indicate positive correlation and blue boxes indicate negative correlation. [file Image_3.tif]

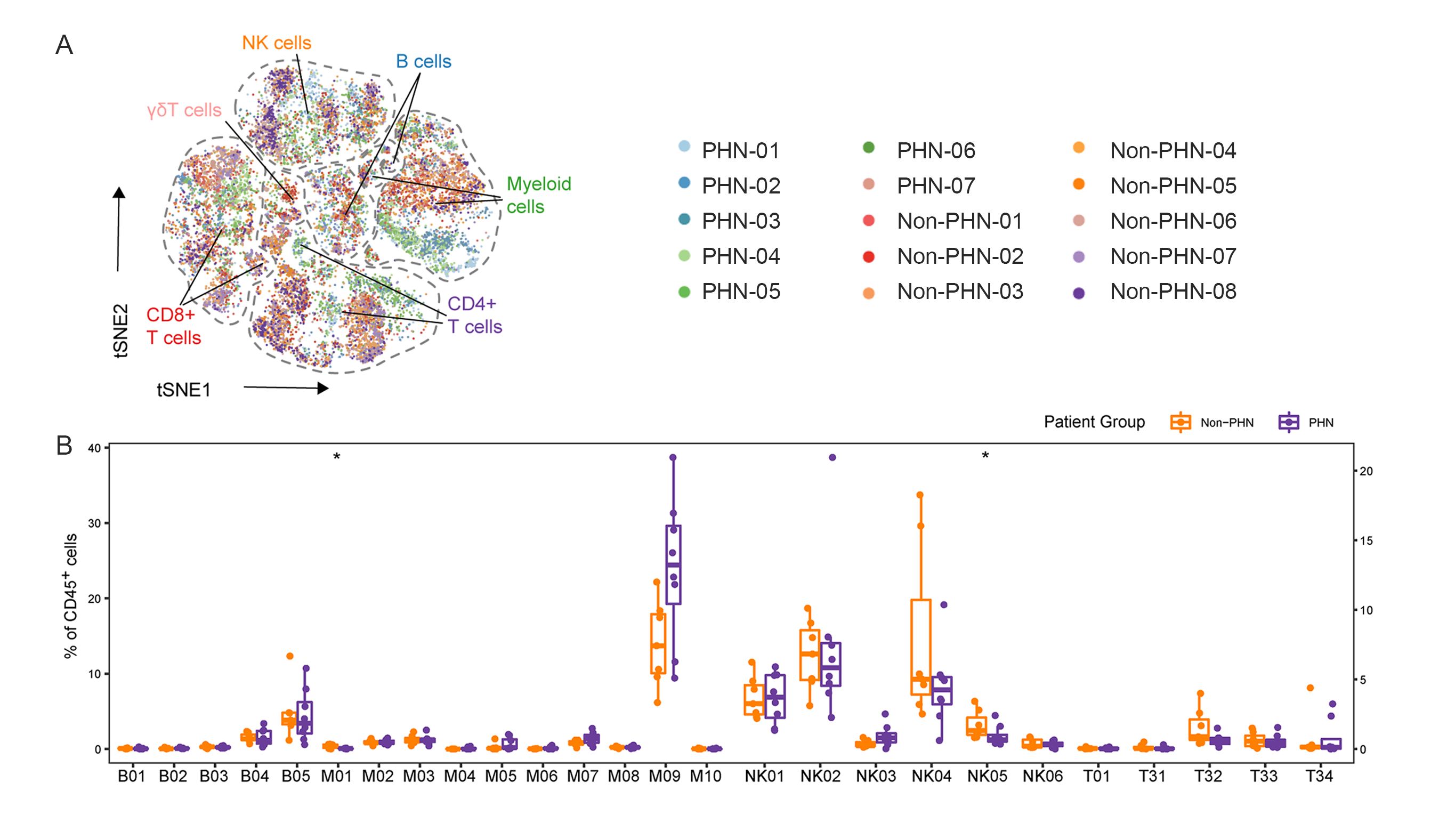

Supplement: Supplementary Figure 4 — The immune landscape of PBMCs in PHN and non-PHN patients (related to Figure 4 ). (A) the merged tSNE plot of each PHN and non-PHN sample. (B) Boxplots exhibiting the frequencies of different phenotypes of B cells, myeloid cells, NK cells, and γδ T cells in PHN and non-PHN groups. [file Image_4.tif]

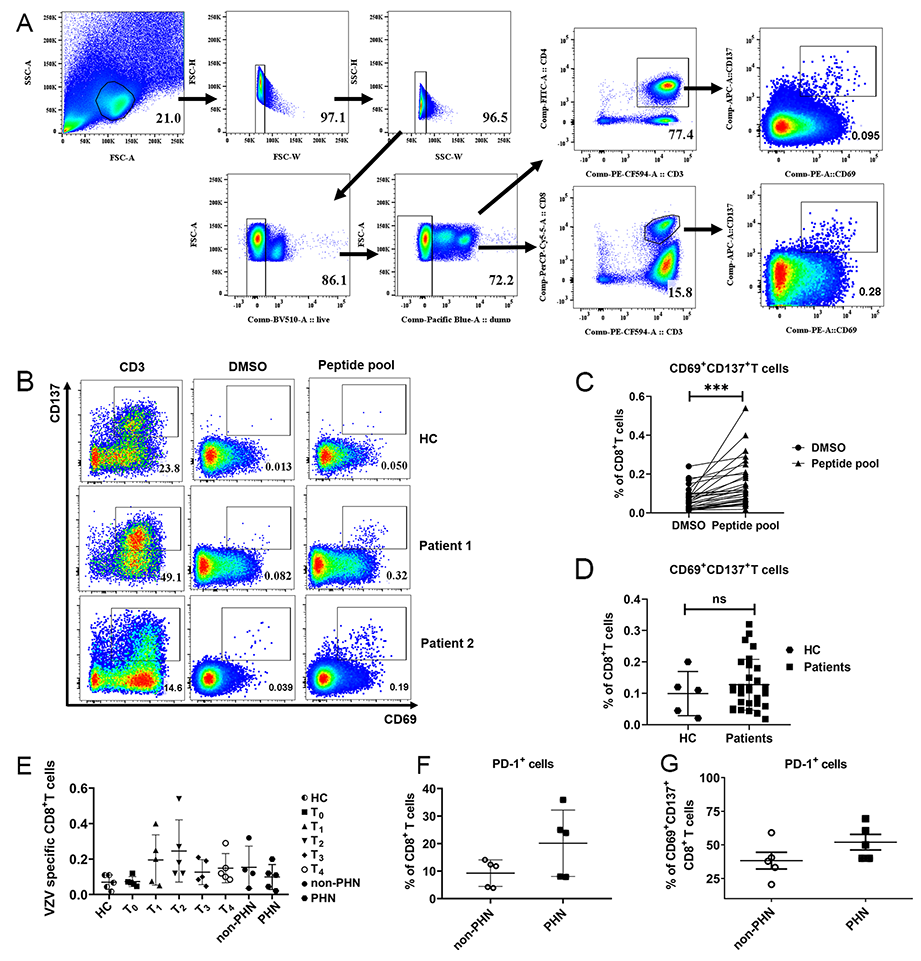

Supplement: Supplementary Figure 5 — The characteristics of VZV specific CD8+ T cell response (related to Figure 5 ). (A) The gating strategy for getting CD69+CD137+CD4+ T cells or CD69+CD137+CD8+ T cells. Dead cells and pacific blue positive cells are excluded for analysis (details in method). (B) Examples displaying the FACS plot, gated on total CD8+ T cells. (C) Comparison of frequency of VZV specific CD8+ T cells in peptide pools or DMSO. (D) Comparison of frequency of VZV specific CD8+ T cells in patients and HC. (E) The characteristics of dynamics of VZV-specific CD8+ T cells in patients at different time point after the onset of rash. (F, G) Percentages of PD-1+ cells in total CD8+ T cells (F) and VZV specific CD8+ T cells (G) at different time point after the onset of rash. [file Image_5.tif]
