# Supplementary material for: Dynamic Immune Landscape and VZV-Specific T Cell Responses in Patients With Herpes Zoster and Postherpetic Neuralgia
Source: Front Immunol. 2022 Jun 1;13:887892. doi: 10.3389/fimmu.2022.887892 (PMC9199063; doi:10.3389/fimmu.2022.887892)
Supplement: Supplementary file 10 [file Table_1.docx]

Supplementary Table 1. Clinical characteristics of the enrolled samples for CyTOF analysis

|  | | **T_0_** | **T_1_** | **T_2_** | **T_3_** | **T_4_ pain-release** | **T_4_ pain** | **non-PHN** | **PHN** | **HC** |
| --- | --- | --- | --- | --- | --- | --- | --- | --- | --- | --- |
| Sample Size | | 8 | 8 | 6 | 7 | 8 | 8 | 7 | 8 | 8 |
| Age (years) | | 56.13±9.55 | 56.13±9.55 | 54.67±8.09 | 57.43±9.59 | 56.13±9.55 | 61.75±8.35 | 55.43±10.24 | 67.5±6.89 | 58.63±14.01 |
| Gender | Female | 5 | 5 | 3 | 4 | 5 | 4 | 5 | 3 | 4 |
|  | Male | 3 | 3 | 3 | 3 | 3 | 4 | 2 | 5 | 4 |
| NRS | | 6.25±2.38 | 4.38±2.07 | 5.00±2.83 | 4.43±1.40 | 0.63±1.19 | 5.50±1.85 | 0.71±0.95 | 3.50±2.73 | - |
| Touch induced pain | | 5.63±1.77 | 4.38±2.00 | 5.83±2.32 | 4.86±1.57 | 0.75±1.16 | 5.38±1.60 | 0.00±0.00 | 3.75±2.82 |  |
| Numbness degree | | 2.25±2.43 | 1.00±1.77 | 3.50±1.05 | 2.00±1.91 | 0.38±1.06 | 3.25±1.91 | 0.43±0.53 | 2.75±2.43 |  |
| DN4 | | 5.25±3.11 | 3.28±2.14 | 4.83±2.48 | 6.00±1.26 | 1.13±1.46 | 6.13±2.03 | 3.00±2.16 | 5.25±2.19 | - |
| ID-pain | | 2.75±1.04 | 2.14±1.57 | 3.00±1.41 | 3.00±1.41 | 1.00±1.31 | 4.38±0.74 | 1.75±0.96 | 3.25±1.16 | - |
| GAD-7 | | 3.50±2.14 | 3.43±4.89 | 4.00±4.98 | 2.80±4.21 | 0.00±0.00 | 6.75±4.43 | 2.50±2.65 | 10.5±15.78 | - |
| PHQ-9 | | 8.75±5.65 | 6.71±5.71 | 5.83±5.23 | 7.80±3.03 | 1.25±3.15 | 12.38±7.42 | 3.25±3.40 | 11.13±7.55 | - |
| Impact of pain on mood | | 5.29±2.43 | 4.00±3.42 | 4.67±2.66 | 5.60±1.82 | 0.00±0.00 | 6.25±3.41 | 0.29±0.49 | 4.38±4.17 | - |
| Impact of pain on daily life | | 5.57±3.55 | 3.75±2.82 | 4.00±3.10 | 1.40±1.34 | 0.13±0.35 | 5.50±2.45 | 0.29±0.49 | 4.38±4.00 | - |

T_0_: onset of skin rash for less than 3 days, before antiviral treatment; T_1_: 1 week after the onset of rash; T_2_: 2 weeks after the onset of rash; T_3_: 3 weeks after the onset of rash; T_4_ pain-release: 4 weeks after the onset of rash with no/durable pain; T_4_ pain: 4 weeks after the onset of rash with undurable pain; non-PHN: 3 months after the onset of rash with no/durable pain; PHN: 3 months after the onset of rash with undurable pain; HC: healthy controls.

Note: The samples of T_0_, T_1_ and T_4_ pain-release represent longitudinal samples from the same patients.
