# Supplementary material for: Dynamic Immune Landscape and VZV-Specific T Cell Responses in Patients With Herpes Zoster and Postherpetic Neuralgia
Source: Front Immunol. 2022 Jun 1;13:887892. doi: 10.3389/fimmu.2022.887892 (PMC9199063; doi:10.3389/fimmu.2022.887892)
Supplement: Supplementary file 11 [file Table_2.docx]

Supplementary Table 2. Clinical characteristics of the enrolled samples for in vitro experiments.

|  | | **T_0_** | **T_1_** | **T_2_** | **T_3_** | **T_4_** | **non-PHN** | **PHN** | **HC** |
| --- | --- | --- | --- | --- | --- | --- | --- | --- | --- |
| Sample Size | | 5 | 5 | 5 | 5 | 5 | 5 | 5 | 5 |
| Age (years) | | 57.60±6.23 | 62.80±1.30 | 58.6±9.29 | 62.00±9.06 | 56.80±17.48 | 56.40±14.19 | 74.80±3.56 | 60.60±11.72 |
| Gender | Female | 4 | 2 | 3 | 3 | 2 | 4 | 2 | 2 |
|  | Male | 1 | 3 | 2 | 2 | 3 | 1 | 3 | 3 |
| NRS | | 1.20±1.10 | 2.20±1.48 | 3.00±0.82 | 1.33±1.53 | 4.60±1.82 | 0.20±0.45 | 0.75±0.95 | - |
| Touch induced pain | | 3.20±3.22 | 4.00±3.39 | 6.50±1.91 | 3.33±3.06 | 4.10±1.60 | 1.00±1.00 | 4.75±3.10 |  |
| Numbness degree | | 2.00±2.55 | 0.00±0.00 | 4.00±4.90 | 0.67±1.15 | 2.60±1.67 | 0.60±0.89 | 1.50±3.00 |  |
| DN4 | | 2.00±1.22 | 3.20±1.64 | 5.50±0.58 | 3.33±2.08 | 6.20±1.30 | 2.60±2.41 | 4.25±2.06 | - |
| ID-pain | | 2.00±1.41 | 2.00±0.71 | 4.25±0.96 | 2.33±1.15 | 4.40±0.89 | 1.00±1.22 | 2.75±0.96 | - |
| GAD-7 | | 4.40±7.70 | 0.80±0.84 | 3.00±3.16 | 3.33±2.89 | 9.20±6.10 | 2.60±3.58 | 9.00±8.21 | - |
| PHQ-9 | | 6.00±9.14 | 2.00±2.92 | 9.00±7.75 | 4.33±4.51 | 10.20±5.54 | 2.80±3.90 | 12.00±11.63 | - |
| Impact of pain on mood | | 1.80±2.95 | 1.20±2.17 | 4.75±1.71 | 3.67±3.21 | 5.60±2.51 | 1.40±1.95 | 5.25±2.87 | - |
| Impact of pain on daily life | | 2.60±2.79 | 1.00±1.73 | 4.75±2.36 | 4.33±2.08 | 6.00±2.12 | 0.40±0.89 | 4.25±3.86 | - |

T_0_: onset of skin rash for less than 3 days, before antiviral treatment; T_1_: 1 week after the onset of rash; T_2_: 2 weeks after the onset of rash; T_3_: 3 weeks after the onset of rash; T_4_: 4 weeks after the onset of rash; non-PHN: 3 months after the onset of rash with no/durable pain; PHN: 3 months after the onset of rash with undurable pain; HC: healthy controls.
