# Supplementary material for: Dynamic Immune Landscape and VZV-Specific T Cell Responses in Patients With Herpes Zoster and Postherpetic Neuralgia
Source: Front Immunol. 2022 Jun 1;13:887892. doi: 10.3389/fimmu.2022.887892 (PMC9199063; doi:10.3389/fimmu.2022.887892)
Supplement: Supplementary file 12 [file Table_3.docx]

Supplementary Table 3. Information of antibodies for CyTOF analysis

| **Reagents** | **Label** | **Source** | **Identifier** |
| --- | --- | --- | --- |
| CD45(HI30)-purified | 89Y | BioLegend | Cat#304002 |
| CD3e(UCHT1)-purified | 115In | BioLegend | Cat#BE0231 |
| CD68(Y1/82A)-purified | 139La | BioLegend | Cat#333802 |
| CD56(NCAM16.2)-purified | 141Pr | BD Biosciences | Cat#559043 |
| TCRδ(5A6.E9)-purified | 142Nd | Homemade |  |
| CD27(O323)-purified | 143Nd | BioLegend | Cat#302802 |
| CD14(M5E2)-purified | 144Nd | BioLegend | Cat#301810 |
| IgD(IA6-2)-purified | 145Nd | BioLegend | Cat#348202 |
| CD123(6H6)-purified | 146Nd | BioLegend | Cat#306002 |
| CD103(B-Ly7)-purified | 147Sm | BioLegend | Cat#14-1038-82 |
| CD19(HIB19)-purified | 148Nd | BioLegend | Cat#333802 |
| CD25(24212)-purified | 149Sm | R&D Systems | Cat#MAB1020 |
| CD57(HCD57)-purified | 150Nd | BioLegend | Cat#322325 |
| Lag3(874501)-purified | 151Eu | R&D Systems | Cat#MAB23193 |
| CD39(A1)-purified | 152Sm | BioLegend | Cat#328202 |
| CD161(HP-3G10)-purified | 153Eu | BioLegend | Cat#339902 |
| CTLA-4(14D3)-purified | 154Sm | BioLegend | Cat#349902 |
| CD45RA(HI100)-purified | 155Gd | BioLegend | Cat#304102 |
| CD24(ML5)-purified | 156Gd | BioLegend | Cat#311102 |
| CD172α/β(SE5A5)-purified | 157Gd | BioLegend | Cat#372102 |
| CD11c(BU15)-purified | 158Gd | BioLegend | Cat#337202 |
| CD45RO(UCHL1)-purified | 159Tb | BioLegend | Cat#304202 |
| CD28(CD28.2)-purified | 160Gd | BioLegend | Cat#BE0291 |
| FoxP3(PCH101)-purified | 162Dy | eBioscience | Cat#14-4776-82 |
| CD33(WM53)-purified | 163Dy | BioLegend | Cat#303419 |
| CD38(HIT2)-purified | 164Dy | BioLegend | Cat#303502 |
| CD69(FN50)-purified | 166Er | BioLegend | Cat#310902 |
| CD278(C398.4A)-purified | 167Er | BioLegend | Cat#313502 |
| T-bet(4B10)-purified | 168Er | BioLegend | Cat#644802 |
| Ki-67(SolA15)-purified | 169Tm | Thermofisher | Cat#14-5698-82 |
| CD127(A019D5)-purified | 170Er | BioLegend | Cat#351302 |
| Eomes(644730)-purified | 171Yb | R&D Systems | Cat#MAB6166 |
| BTLA(MIH26)-purified | 172Yb | BioLegend | Cat#344504 |
| GranzymeB(GB11)-purified | 173Yb | Fluidigm | Cat#3173006B |
| PD-1(EH12.2H7)-purified | 174Yb | BioLegend | Cat#329926 |
| CD16(3G8)-purified | 175Lu | BioLegend | Cat#302014 |
| HLA-DR(L243)-purified | 176Yb | BioLegend | Cat#307612 |
| CD4(RPA-T4)-purified | 197Au | BioLegend | Cat#300516 |
| CD8(RPA-T8)-purified | 198Pt | BioLegend | Cat#301018 |
| CD11b(M1/70)-purified | 209Bi | BioLegend | Cat#101202 |
| CD66b(6/40c)-purified | 165Ho | BioLegend | Cat#392902 |
| ProMBP1(J175-7D4)-purified | 161Dy | BioLegend | Cat#346802 |
