# Supplementary material for: Dynamic Immune Landscape and VZV-Specific T Cell Responses in Patients With Herpes Zoster and Postherpetic Neuralgia
Source: Front Immunol. 2022 Jun 1;13:887892. doi: 10.3389/fimmu.2022.887892 (PMC9199063; doi:10.3389/fimmu.2022.887892)
Supplement: Supplementary file 13 [file Table_4.docx]

Supplementary table 4: The phenotype of each cluster

| Cluster | Phenotype | Cluster | Phenotype |
| --- | --- | --- | --- |
| B01 | IgD^+^CD27^-^CD38^-^CD19^+^ | T08 | CD27^+^CD45RA^-^CD4^+^ |
| B02 | IgD^-^CD27^-^CD38^-^CD19^+^ | T09 | CD27^+^CD45RA^low^CD4^+^ |
| B03 | IgD^-^CD27^+^CD38^+^CD19^+^ | T10 | CD28^+^CD45RO^+^CD4^+^ |
| B04 | IgD^-^CD27^low^CD38^-^CD19^+^ | T11 | CD27^-^CD57^-^GranzymeB^+^Tbet^+^CD4^+^ |
| B05 | IgD^+^CD27^-^CD38^+^CD19^+^ | T12 | CD27^-^CD57^+^CD45RA^+^CD4^+^ |
| M01 | CD16^low^CD14^-^CD11b^low^HLA-DR^+^ | T13 | CD25^+^FOXP3^+^CD45RO^+^CD4^+^ |
| M02 | CD123^+^CD11c^low^ CD33^low^CD11b^low^ | T14 | CD27^low^CD45RO^+^CD4^+^ |
| M03 | HLA-DR^+^CD33^+^CD11c^+^CD11b^low^CD172a^+^ | T15 | GranzymeB^+^Tbet^+^CD45RO^+^CD4^+^ |
| M04 | HLA-DR^low^CD33^low^CD11c^+^CD11b^low^ CD172a^low^ | T16 | GranzymeB^+^Tbet^+^CD45RO^+^CD4^+^ |
| M05 | CD16^+^ CD14^-^CD11b^+^ | T17 | CD161^+^CD45RO^+^CD4^+^ |
| M06 | CD69^+^CD11b^low^CD68^+^ | T18 | BTLA^+^CD27^+^CD127^+^CD57^low^CD8^+^ |
| M07 | CD123^low^CD11c^+^CD11b^low^HLA-DR^+^CD33^low^ | T19 | CD27^low^CD103^low^CD45RO^+^CD8^+^ |
| M08 | CD123^+^ HLA-DR^+^CD68^+^ | T20 | PD-1^+^CD45RO^+^CD8^+^ |
| M09 | CD16^-^CD14^+^CD11b^+^ | T21 | CD57^+^CD45RO^+^CD38^low^CD8^+^ |
| M10 | CD161^+^CD25^+^CD11b^low^CD11c^low^ | T22 | CD27^low^CD45RO^low^CD69^-^GranzymeB^low^CD8^+^ |
| NK01 | CD56^high^CD57^-^CD161^+^ | T23 | GranzymeB^+^Tbet^+^CD57^+^CD45RO^low^CD8^+^ |
| NK02 | CD56^low^CD16^+^ | T24 | GranzymeB^low^Tbet^low^CD57^+^CD45RO^low^CD8^+^ |
| NK03 | CD56^low^CD57^-^CD161^low^ | T25 | CD27^low^CD45RO^low^CD8^+^ |
| NK04 | CD56^low^CD57^+^CD16^-^ | T26 | CD27^+^CD57^-^CD8^+^ |
| NK05 | CD56^high^CD16^-^ | T27 | CD45RA^+^CD27^-^CD28^-^CD69^-^CD8^+^ |
| NK06 | CD56^high^CD16^-^ | T28 | CD161^+^CD45RO^+^CD8^+^ |
| T01 | CD4^+^CD8^+^ | T29 | CD56^+^CD57^+^CD45RO^+^CD38^+^CD8^+^ |
| T02 | BTLA^+^CD27^+^CD127^+^CD57^low^CD4^+^ | T30 | CD69^+^HLA-DR^+^CD8^+^ |
| T03 | CD127^high^CD45RO^+^CD4^+^ | T31 | CD45RO^+^CD27^-^TCRδ^+^ |
| T04 | CD27^+^CD45RA^+^CD38^+^CD4^+^ | T32 | CD45RO^+^CD27^+^TCRδ^+^ |
| T05 | CD27^-^CD57^+^CD45RO^+^CD4^+^ | T33 | CD45RO^-^TCRδ^+^ |
| T06 | CD27^-^CD57^+^CD45RO^low^CD56^-^CD4^+^ | T34 | CD45RO^low^TCRδ^+^ |
| T07 | CD27^-^CD57^+^CD45RO^low^CD56^+^CD4^+^ |  |  |
